# RETRACTION: Correlation of Integrin Alpha 7 With Clinicopathological Characteristics and Survival Profiles, as Well as its Regulatory Role in Cell Proliferation, Apoptosis, and Stemness in Non‐Small‐Cell Lung Cancer

**DOI:** 10.1002/jcla.70361

**Published:** 2026-09-22

## Abstract

**RETRACTION**: D. Xia, B. Chen, X. Yang, “Correlation of Integrin Alpha 7 With Clinicopathological Characteristics and Survival Profiles, as Well as its Regulatory Role in Cell Proliferation, Apoptosis, and Stemness in Non‐Small‐Cell Lung Cancer,” *Journal of Clinical and Laboratory Analysis* 33, no. 8 (2019): e22973, https://doi.org/10.1002/jcla.22973.

The above article, published online on 16 August 2019 in Wiley Online Library (wileyonlinelibrary.com), has been retracted by agreement between the journal Editor‐in‐Chief, Rong Fu; and Wiley Periodicals, LLC. The retraction has been agreed due to concerns raised by a third party that identified indications of compromised data and systematic issues within the published article, including inconsistencies related to patient recruitment. Furthermore, a panel in Figure 1A was found to have been used in another article in a different scientific context. The authors did not provide the requested documentation relating to ethics approval, patient informed consent, or the underlying raw data. Accordingly, the article is retracted as the editors have lost trust in the reliability of the data and the results presented. The authors have been informed of this decision but were not available for final confirmation.

**RETRACTION**: D. Xia, B. Chen, X. Yang, “Correlation of Integrin Alpha 7 With Clinicopathological Characteristics and Survival Profiles, as Well as its Regulatory Role in Cell Proliferation, Apoptosis, and Stemness in Non‐Small‐Cell Lung Cancer,” *Journal of Clinical and Laboratory Analysis* 33, no. 8 (2019): e22973, https://doi.org/10.1002/jcla.22973.

The above article, published online on 16 August 2019 in Wiley Online Library (wileyonlinelibrary.com), has been retracted by agreement between the journal Editor‐in‐Chief, Rong Fu; and Wiley Periodicals, LLC. The retraction has been agreed due to concerns raised by a third party that identified indications of compromised data and systematic issues within the published article, including inconsistencies related to patient recruitment. Furthermore, a panel in Figure 1A was found to have been used in another article in a different scientific context. The authors did not provide the requested documentation relating to ethics approval, patient informed consent, or the underlying raw data. Accordingly, the article is retracted as the editors have lost trust in the reliability of the data and the results presented. The authors have been informed of this decision but were not available for final confirmation.

